# 3dRNA v2.0: An Updated Web Server for RNA 3D Structure Prediction

**DOI:** 10.3390/ijms20174116

**Published:** 2019-08-23

**Authors:** Jun Wang, Jian Wang, Yanzhao Huang, Yi Xiao

**Affiliations:** School of Physics and Key Laboratory of Molecular Biophysics of the Ministry of Education, Huazhong University of Science and Technology, Wuhan 430074, China

**Keywords:** RNA structure prediction, scoring function, structure optimization

## Abstract

3D structures of RNAs are the basis for understanding their biological functions. However, experimentally solved RNA 3D structures are very limited in comparison with known RNA sequences up to now. Therefore, many computational methods have been proposed to solve this problem, including our 3dRNA. In recent years, 3dRNA has been greatly improved by adding several important features, including structure sampling, structure ranking and structure optimization under residue-residue restraints. Particularly, the optimization procedure with restraints enables 3dRNA to treat pseudoknots in a new way. These new features of 3dRNA can greatly promote its performance and have been integrated into the 3dRNA v2.0 web server. Here we introduce these new features in the 3dRNA v2.0 web server for the users.

## 1. Introduction

Noncoding RNAs can catalyze and regulate many biochemical reactions in organisms [1]. Just like proteins, to understand these functions, it is important to know their 3D structures. However, the number of solved RNA 3D structures is still quite small compared with the large amount of non-coding RNA sequences and this becomes an obstacle to explore their functions. Therefore, many computational methods have been proposed for predicting the 3D structure of non-coding RNA, e.g., FARNA [2]/FARFAR [3], MC-Sym [4], NAST [5], iFoldRNA [6,7], ASSEMBLE [8], Vfold [9,10], RNAComposer [11,12], 3dRNA [13,14,15] and F-RAG [16].

3dRNA is an automated method of building RNA 3D structures from sequences and secondary structures by using the smallest secondary elements (SSEs) [14]. The SSEs are defined as stem (helix), hairpin loop, internal loop, bulge loop, pseudoknot loop and junction loop. 3dRNA has been used by many researchers in practice [16,17,18,19,20,21,22,23]. Up to now, it has performed over 5500 tasks from other groups and was visited by more than 6000 individual IP addresses. The 3dRNA v2.0 web server can be accessed at http://biophy.hust.edu.cn/3dRNA.

## 2. Results

**Figure 1:**
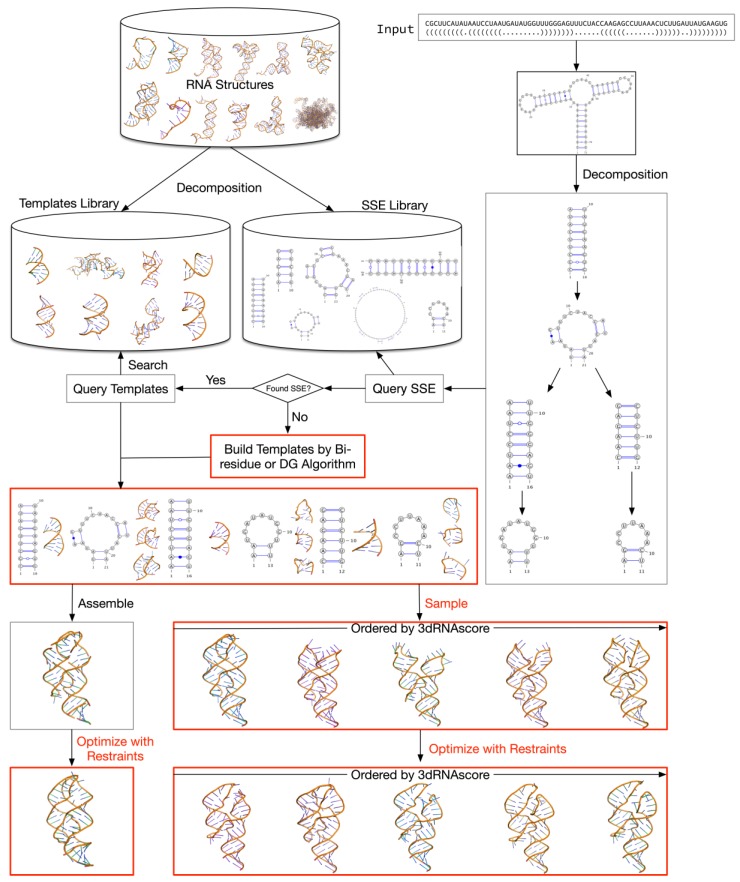
Schematic diagram of the prediction process of an RNA with pseudoknot (PDB ID: 1Y26 [30]) using 3dRNA v2.0. The red colored parts of this diagram are the new features compared with our previous web server.

Our original 3dRNA web server can only do one type of task: users just need to input the sequence and secondary structure of an RNA and click the “submit” button, after which they can obtain its predicted structures automatically [14,15]. Recently, we have extended 3dRNA to include a method to further optimize the predicted 3D structures using the restraints obtained from experiments or theoretical predictions [24]. This optimization method can greatly increase the accuracy of the predicted structures using 3dRNA, as well as other RNA 3D structure prediction methods [24]. Furthermore, we have also implemented an algorithm of direct coupling analysis (DCA) [25] to find the most likely contacted nucleotide pairs that can be used as the restraints in the optimization procedure. In particular, the optimization method enables 3dRNA to treat pseudoknots in a new way. Instead of finding 3D templates for pseudoknots, 3dRNA takes the base pairs in the pseudoknots as restraints and then optimizes the predicted initial RNA 3D structures with the restraints (see the right bottom of Figure 1). It avoids the problem of missing templates for pseudoknots. On the other hand, since sampling and optimization processes usually are time-consuming, it is better to let users determine whether they need these processes or not. We have greatly modified the 3dRNA web server to incorporate these new features into it, which are colored red in Figure 1, and make it easy to use.

### 2.1. 3dRNA

Here we briefly describe the main steps of 3dRNA. Detailed descriptions of them can be found in our previous papers [13,14,15,24], and they can be elucidated by the schema diagram in Figure 1. Firstly, we decompose an RNA secondary structure into a tree, each node of which corresponds to an SSE and each edge connects two successive SSEs. Secondly, we search the template library and obtain a suitable 3D template for each of the nodes. Thirdly, we assemble the selected template of each node with that of its parent node. The Kabsch method [26] is used as the superposition method. After traversing through the tree, we can get a complete tertiary structure. For those nodes (SSE) that have more than one 3D template, the template having the highest sequence homology with the query SSE will be selected firstly. If users want to get more than one prediction, other templates will be selected randomly. In the case of missing 3D templates for a specific SSE, 3dRNA will use a bi-residues method (see Section 4) or distance-geometry method [27,28] to generate its 3D templates. As an option, users of the 3dRNA web server can generate 3D templates for all SSEs of an RNA using these two methods and use them to assemble the RNA structure instead of the 3D templates extracted from the experimental structures. In the final step, 3dRNA clusters the candidates using the k-means algorithm and then uses the 3dRNAscore [13] to evaluate the centroids of each cluster. Then, users are free to choose the appropriate structures from them or feed them to the optimization procedure with addition of restraints from experiments and/or theoretical predictions [24]. It is noted that 3dRNA has no size limitation, though the accuracy will become lower for longer junctions.

The SSE 3D templates library has been updated. It is divided into two major sub-libraries: helix library and loop library. The helix library includes all kinds of helices extracted from experimentally determined RNA structures of different lengths and different sequences. Usually, there is more than one template for an SSE. The loop library consists of different types of loops including hairpin loops, bulge loops, internal loops and multi-branch loops. Two base pairs are attached to the ends of every loop, thus we can assemble two successive templates more accurately by overlapping them. Hairpin loops, bulge loops and internal loops involve all available types and sequences. The multi-branch loops (or named junction) contain 3-way junction, 4-way junction, 5-way junction, and so on. Currently, there are 27,163 helices and 27,826 loops in the library.

It has been shown that the prediction accuracy of 3dRNA and other widely used RNA 3D structure prediction methods could be improved significantly by the restraints from coevolutionary information obtained using direct coupling analysis (DCA) [24]. For instance, the averaged prediction accuracy is 9.61 Å and 8.05 Å for a small test set which contains 5 short RNAs (78 nt in average, PDB (Protein Data Bank) ID: 1FIR [29], 1Y26 [30], 2GDI [31], 3Q3Z [32], 4LVV [33]), with or without the DCA restraints, respectively. Particularly, nearly all these RNAs (except 2GDI) have pseudoknot, which is always a problem for RNA 3D structure predictions.

**Figure 2:**
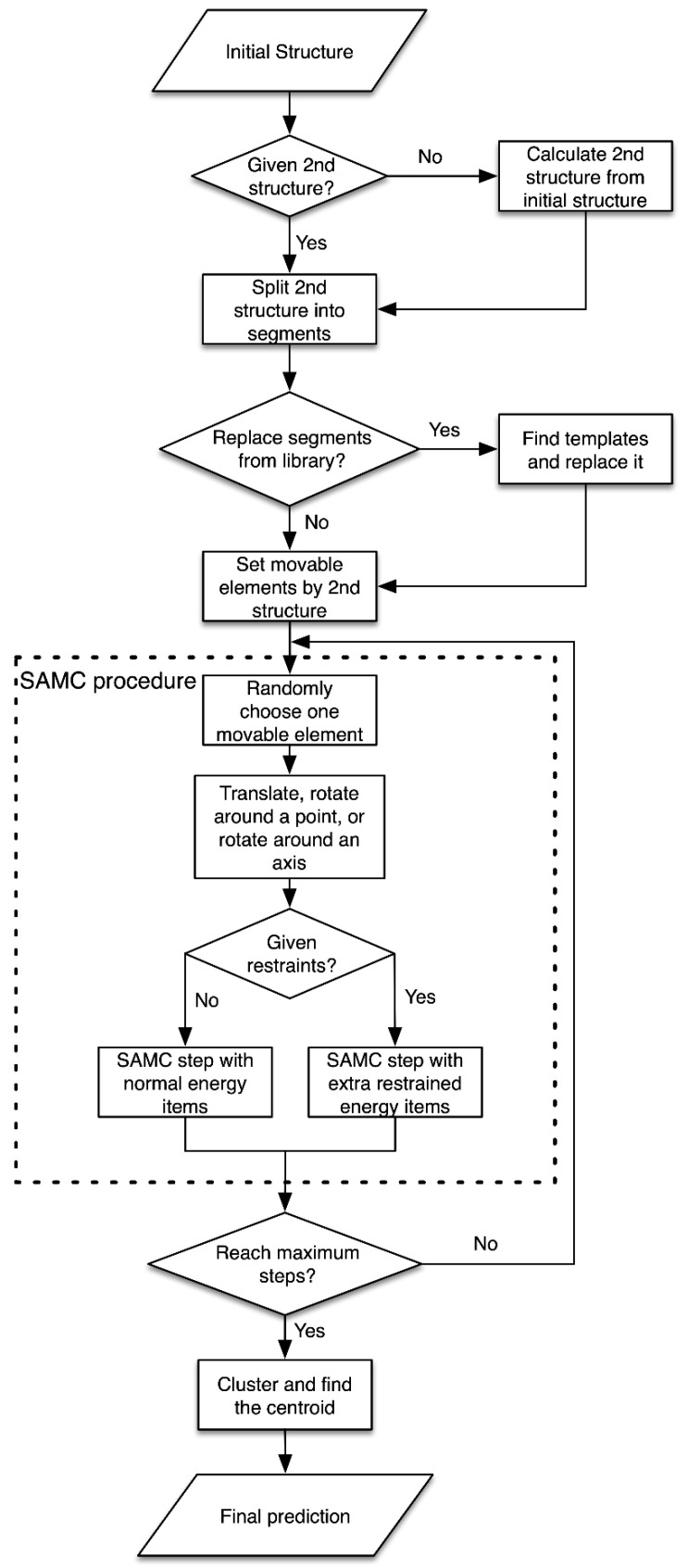
The flowchart of optimization procedure in 3dRNA. SAMC—simulated annealing Monte Carlo sampling method.

The schema diagram of the optimization procedure is shown in Figure 2 and the details can be found in our previous paper [24]. Given the initial RNA structure, 3dRNA will set the movable elements of it according to the secondary structure, which may be given by the user or calculated from the initial structure. The movable elements are all possible fragments that do not interrupt any helices, which keeps the secondary structure unchanged during optimization. In the simulated annealing Monte Carlo (SAMC) optimization process, a randomly chosen moveable element will be translated, rotated around a point, or rotated around an axis. If there are any restraints (DCA or distance), the optimization process can be done under them. Then, a set of conformations are sampled and clustered by using the k-means clustering algorithm according to their Root-Mean-Square-Deviation (RMSD) values from each other. The centroid of each cluster is determined and ranked by 3dRNAscore [13]. Finally, the ranked top N predictions are given to the user. It is worth noting that this clustering step is valuable as it can produce final predictions which are dissimilar to each other. All of these routines have been integrated into our new web server.

### 2.2. User Interface

**Figure 3:**
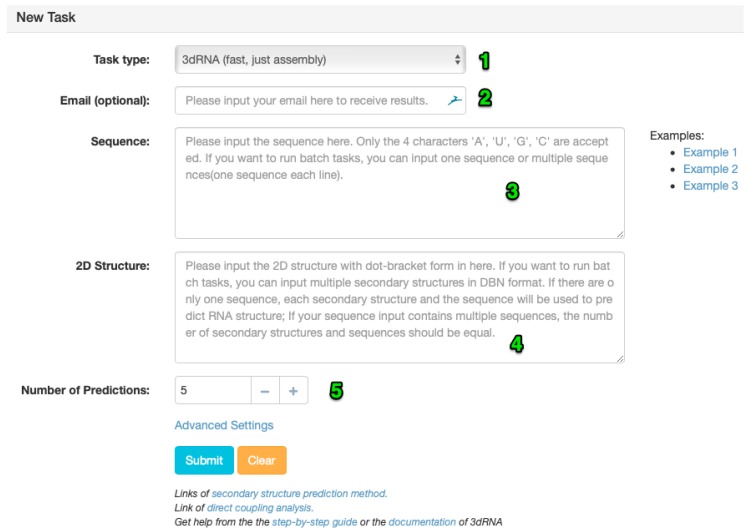
The main page of the 3dRNA web server. The fields labelled with a number in the figure are as follows: (1) “Task Type” field is used to select one of the task types in the following section. (2) The email you want to use to get notification after the job is finished; (3) A “Sequence” field is a required field, which is the sequence of the RNA you want to predict. (4) The “2D structure” field is the corresponding secondary structure for the sequence. (5) The “Number of Predictions” field is used to specify the number of final predictions you want to get.

3dRNA web server has a main page that contains a new task area, a task query area, and an area for references. The UI of the new task area is shown in Figure 3. Most of the time, you will just need to input the sequence and secondary structure of an RNA and the type of task you are going to run, though several advanced options are available when you want to customize your prediction, such as the loop building method and the excluded PDB IDs in the circumstance that you want to validate and test our prediction method. Users can directly use 3dRNA without registering. The main change of the 3dRNA web server is in the new task area, where users are now free to choose different “Task type”.

There are four types of tasks:

1. **3dRNA (fast, just assembly)**: This is the most basic task of 3dRNA. It just finds 3D templates for each SSE and then assembles one or more sets of templates together as the final predictions. The number of assembled structures, N, is determined by the number of predictions set by users (the default is 5). Among the N assembled structures, the first one is assembled using the top-ranked template of each SSE, which has the same secondary structure and most similar sequence with that of the query. The other N-1 assembled structures are assembled from randomly chosen templates of each SSE. The assembled structures will be refined through an energy minimization procedure using AMBER [34] in default. Therefore, this type of task is fast in speed.
2. **3dRNA with sampling (medium, assembly → sampling)**: For this type of task, 3dRNA firstly builds a structure by assembling the top-ranked template of each SSE and then samples different templates for each of them. At each sampling step, the 3D structure of each SSE is replaced by a randomly chosen candidate from available templates for the SSE in order to increase the diversity of predictions. Users are able to set the number of sampling steps (the default is 500). After that, the sampled structures are clustered by the analytical routine which uses a k-means algorithm in 3dRNA, and the centroids of all clusters are evaluated by our 3dRNAscore program. The number of clusters is determined by the “Number of Predictions” field set by users (the default is 5). Similarly, the finally chosen structures will be refined using the energy minimization procedure of AMBER by default. Obviously, this type of task is slower than the one above.
3. **3dRNA with optimization (slow, assembly → optimization)**: In this type of task, 3dRNA firstly assembles 3D structures in the same way as in task (1), and then it optimizes each of the assembled structures [24]. Users are capable of running the optimization procedure with or without restraints. There are two kinds of restraints acceptable: base pairs (like those given by DCA) or nucleotide-nucleotide distances. The DCA information is off-the-shelf from our DCA web server (http://biophy.hust.edu.cn/DCA). Inside our server, the inputted RNA pseudoknots in dot-bracket format are converted to restraints automatically. Therefore, to predict the 3D structure of an RNA with pseudoknots, you need to choose task type “3dRNA with optimization (slow, assembly → optimization)”. The speed of the optimization procedure is much slower than that of the above two types of tasks. It takes about 30 min to optimize a 75-nt-long RNA on our server, which has an Intel(R) Xeon(R) CPU E5620 @ 2.40 GHz CPU. However, our optimization method shows an approximately linear relationship between the running time and the sequence length. For example, for an RNA of about 400 nucleotides, the overall time of the optimization procedure is about 100 min [24].
4. **Optimization**: In this case, the optimization procedure is a standalone function for optimizing a given RNA structure. Users are capable of optimizing their uploaded RNA structures in PDB format, with or without the restraints described above.

### 2.3. Cases

**Figure 4:**
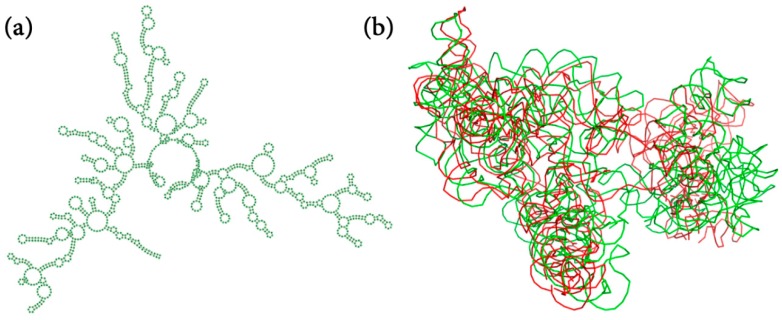
The secondary structure (**a**) and predicted 3D structure (**b**) of 4ADV (1410 nucleotides). The native and predicted structures are in green and red, respectively.

3dRNA has a particular advantage in predicting long RNAs after the addition of the optimization procedure. Take the ribosomal RNA (PDB ID: 4ADV) with a length of 1410 nucleotides [35] as an example. The all-atom RMSD of the best predicted structure is 36.9 Å, and the Interaction Network Fidelity (INF) of canonical Watson‒Crick base pairs and base stacking are 0.91 and 0.81, respectively. Figure 4a shows the complicated 2D structure of 4ADV plotted by Forna [36], and Figure 4b shows the predicted and native 3D structure of it. The overall shape of the predicted structure is similar to the experimental one. Other examples can be found in ref [24]. 3dRNA v2.0 can predict 3D structures of RNAs with arbitrary size and any complicated topology, although the prediction accuracy needs further improvement.

**Figure 5:**
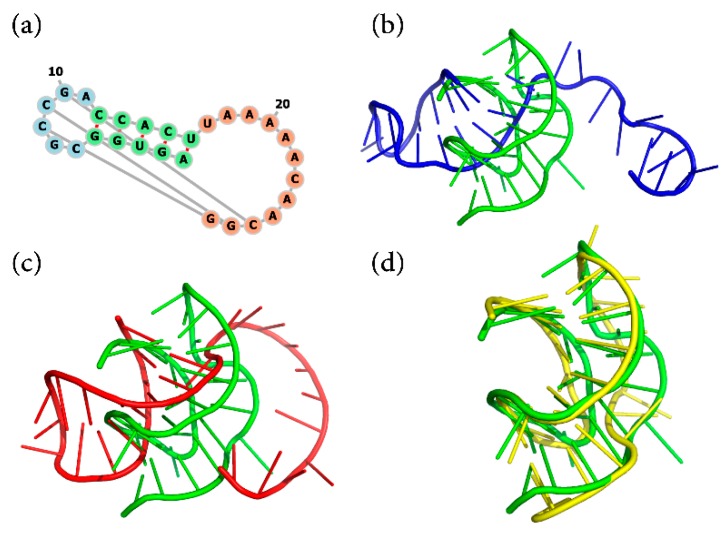
The secondary structure and predicted 3D structures for an RNA (PDB ID 2AP5): (**a**) is its secondary structure, (**b**) is the assembled structure (blue), (**c**,**d**) are the optimized structures not using (red) and using (yellow) the pseudoknot interactions as restraints, respectively. The native structure is represented in green cartoon.

As mentioned above, 3dRNA v2.0 treats pseudoknots using optimization under restraints. Figure 5 shows an example, the frameshift stimulating hairpin-type mRNA pseudoknot (PDBID: 2AP5, 28 nucleotides) [37]. The secondary structure of it is displayed in Figure 5a, and the predicted structures are shown in Figure 5b–d, respectively. For this RNA with pseudoknot interactions, our optimization without restraints can only slightly reduce the RMSD of the assembled structure relative to native structure from about 13.2 Å to 12.2 Å. However, by taking pseudoknot as restraints, the optimization can decrease the RMSD value hugely from about 12.2 Å to 5.9 Å, which can be seen clearly from Figure 5c,d. The INF of the canonical Watson‒Crick base pairs and base stacking of the final predicted structure are 0.63 and 0.67, respectively.

### 2.4. Server Architecture

In order to facilitate the users to do a lot of predictions using our web server. Our web server can do RNA 3D structure predictions in batch mode now. Two modes are accepted—one sequence with different secondary structures and multiple sequences with the same number of secondary structures in DBN (dot bracket notation) format. In the former mode, you should input only one sequence in the field labelled number “3” in Figure 3 and multiple secondary structure strings (one for each line). The backend of our server will do predictions for each of these secondary structures. In the later mode, the RNA sequences and secondary structures in the field labelled number “3” and “4” should contain the same number of lines. Each line of sequence and the corresponding line of secondary structure will be used to do predictions. After the batch job is submitted successfully, the task IDs will be shown in a dialog. If you provide an email, the results will be sent to you after the job is finished.

The web server is based on Linux, Nginx, MySQL and PHP (LNMP) and built on a Laravel framework (https://laravel.com). The frontend is created using HTML5, CSS3, vue.js, JavaScript and Ajax, and the backend job manager is SLURM (https://www.schedmd.com/). The predicted structures are displayed using Jmol in the result page (http://www.jmol.org/).

## 3. Discussion

The large RMSD of the predicted structures for large and complex RNA may stem from two reasons. The first one is the inaccuracy or lack of SSE templates, especially for multi-branch junctions; the second one is the absence of long-range tertiary interaction information. The former pitfall affects the spatial arrangement of SSE templates connected to the multi-branch junctions directly, and the latter one leads to the ignorance of some important restraints that may greatly improve the prediction accuracy. Besides this, the accuracy of RNA secondary structure prediction also significantly affects that of 3D structure prediction [38]. Therefore, in order to solve the problem, we may move along these directions in the future.

## 4. Materials and Methods

### 4.1. Prediction Methods

The predicted structures are built from the sequence and secondary structure, while the former is obtained from their native structures fetched from PDB (https://www.rcsb.org/), and the latter is calculated from DSSR (Dissecting the Spatial Structure of RNA) [39]. In the assembling process, which is able to assemble structures from sequence and secondary structure using templates from solved native structures, the templates that come from the same PDB ID are excluded to test the general performance of 3dRNA. In order to get the multiple sequence alignment from the target sequence, the Infernal package [40] is used. The DCA results are calculated using our DCA web server (http://biophy.hust.edu.cn/DCA) and only preserve the top N values, while the N is calculated by multiplying the RNA sequence length and the factor 0.2. All initial structures that are the start points of optimization are attained by the assembling procedure in 3dRNA.

### 4.2. Simulated Annealing Monte Carlo Simulation

At each step of the optimization process, we choose one movable element randomly, which is determined by the secondary structure of RNA. Then, the translation procedure or rotation procedure is applied to the selected element. After that, the energy of the new conformation is calculated, and the Metropolis algorithm is used to determine whether this moving is accepted or rejected. However, sometimes the structures are trapped in local minima and the traditional Monte Carlo simulation is not enough to overcome the potential barrier. Thus, we use the simulated annealing Monte Carlo (SAMC) simulation method to optimize RNA structures. The simulated annealing protocol is composed of two steps: firstly, the simulated system is heated from low temperature to high temperature; secondly, the simulated system is cooled from high temperature to low temperature. This two-step process is easy to implement than the traditional Monte Carlo simulation procedure and usually improves the sampling efficiency.

### 4.3. The k-Means Clustering Method

After the optimization procedure, there will be a large amount of conformations. In order to get more than one representative structure from this ensemble, we use the k-means clustering method. The number of final predictions you want to get is the number of clusters (say the k super parameter in k-means). The distance for a pair of conformations is the RMSD between them. In a k-means clustering algorithm, there is a method to determine the mean point for each cluster. In our program, the mean point in each cluster is the one with the smallest sum of distance to all other points, which can be represented by the formula below:

$$s_{i}=\underset{k}{\arg\min}\overset{c_{i}}{\underset{j,j\neq i}{\sum }}d_{k,j}$$

where $c_{i}$ is the number of points in ith cluster, j,k is the index of points, and $d_{k,j}$ is the distance between jth and kth point. $s_{i}$ is the centroid of the ith cluster.

### 4.4. Bi-Residue Method

The bi-residue method is used to generate 3D loop motifs of arbitrary types and it works as follows: (i) break the 5S ribosome RNA structure into segments of two continuous nucleotides (bi-residue templates); (ii) build an initial chain of the SSE by randomly choosing templates from these segments; (iii) replace the bi-residues in the chain in random position by randomly choosing a bi-residue template; (iv) accept or reject the replacement using the Metropolis algorithm, and after a number of steps, the template of the SSE is generated.

### 4.5. Distance Geometry

The distance-geometry (DG) method is used to generate 3D loop motifs of arbitrary types and it works as follows: we first get statistics of distances between atoms within a nucleotide, between adjacent nucleotides, between paired nucleotides and between stacked nucleotides, respectively, from the known RNA 3D structures in the PDB. From these statistics we can determine the upper and the lower limits of these distances. Then, we can generate the distances between atoms of the residues for a given loop, which are between the corresponding upper and lower limits. Finally, the 3D coordinates can be obtained using the EMBED algorithm utilized in DG [27,28].

## 5. Conclusions

We have added new features in our 3dRNA web server v2.0, including the sampling procedure and optimization procedure. All users are free to submit their tasks according to their needs. The input of our web server is easy to get and understand, and the running time for different tasks is reasonable. Moreover, the predicted results are reliable, and the UI is user friendly.
